# NCTD Prevents Renal Interstitial Fibrosis via Targeting Sp1/lncRNA Gm26669 Axis

**DOI:** 10.7150/ijbs.59195

**Published:** 2021-07-25

**Authors:** Jiao Tian, Zheng Xiao, Ju Wei, Yi Shan, Dong Zeng, Yilin Tao, Xi Fang, Chengyuan Tang, Xiaojun Chen, Ying Li

**Affiliations:** 1 Department of Nephrology, The Second Xiangya Hospital, Central South University, Changsha, Hunan 410011, China.; 2 Key laboratory of kidney Disease and Blood Purification in Hunan Province, Changsha, Hunan 410011, China.

**Keywords:** norcantharidin, special protein 1, lncRNA Gm26669, nuclear translocation, renal interstitial fibrosis

## Abstract

In our previous study, we demonstrated that norcantharidin (NCTD) is a potential therapeutic agent for renal interstitial fibrosis (RIF). Recently, we found that lncRNA Gm26669 (Gm26669) contributed to the development of RIF and could be regulated by NCTD. However, the upstream mechanisms of Gm26669 and whether the anti-RIF effects of NCTD are related to its regulatory action on Gm26669 remain unclear. Our bioinformatics analysis indicated that special protein1 (Sp1), a transcription factor, may bind to the promoter of Gm26669. In the present study, we observed a significant increase in the nuclear translocation of Sp1 using both *in vivo* and *in vitro* models of RIF. Furthermore, the knockdown of Sp1 inhibited the expression of collagen type I (CoL-I) and fibronectin (Fn). Mechanistically, Sp1 promoted the expression levels of CoL-I and Fn by directly binding to the promoter of Gm26669 to elevate its expression level. Moreover, we found that NCTD alleviated RIF by inhibiting Gm26669 and the nuclear translocation of Sp1. Collectively, above results suggested that NCTD might prevent RIF via targeting the Sp1/Gm26669 axis, thus providing a new theoretical basis for the clinical application of NCTD in the treatment of RIF.

## Introduction

Chronic kidney disease (CKD) has become a major global public health problem. In 2017, the global burden of disease statistics showed that the prevalence of CKD was 9.1% 1. Renal interstitial fibrosis (RIF) is a common pathological feature of progressive injury in CKD and predominantly includes renal tubular epithelial cell atrophy, inflammatory cell infiltration, and the excessive deposition of extracellular matrix (ECM) 2, 3. At present, the main clinical drugs administered to delay the progression of RIF are angiotensin converting enzyme inhibitors (ACEIs) and angiotensin receptor antagonists (ARBs); however, these drugs are limited by certain clinical side effects that lead to residual damage to renal function 4. Therefore, there is a significant need to identify novel and specific drugs for the treatment of RIF.

Norcantharidin (NCTD), a synthetic derivative of cantharidin (a form of traditional Chinese medicine) is widely used for anti-tumor therapy 5. Compared with cantharidin, NCTD does not inhibit the production of bone marrow cells or leukocytes, exhibits less nephrotoxicity, and enhances immunity in patients 6, 7. Our previous studies demonstrated that NCTD could effectively prevent RIF both *in vivo* and *in vitro* 8-11. Furthermore, we confirmed that NCTD prevented RIF via inhibiting the dephosphorylation of β-catenin and phosphorylation of Smad3 7, 12. However, the underlying mechanism responsible for the action of NCTD on RIF has yet to be fully elucidated.

Long non-coding RNAs (lncRNAs) are transcripts that contain more than 200 nucleotides but have a limited ability to encode proteins 13. Recent studies have shown that lncRNAs play critical roles in RIF 14, 15. According to the results of previous RNA-sequencing (RNA-Seq) analysis, lncRNA Gm26669 (Gm26669) was upregulated in the kidneys of a mouse model of unilateral ureteral obstruction (UUO) and was downregulated following treatment with NCTD 16. In addition, we have demonstrated that the overexpression of Gm26669 promoted RIF and the knockdown of Gm26669 attenuated RIF in UUO mice, and in TGF-β1-stimulated BUMPT cells (unpublished data). However, the upstream mechanisms of Gm26669 and whether the anti-RIF effects of NCTD are related to its regulatory action on Gm26669 remain unclear.

It has been reported that the expression of lncRNA is usually regulated by transcription factors 17. We used the PROMO database to predict the transcription factors that might bind to the promoter of Gm26669 and found that 97 transcription factors may undergo interactions with the promoter of Gm26669. In addition, RNA-Seq from three groups of kidney tissues (sham, UUO, and NCTD groups) identified that 113 transcription factors may be regulated by NCTD. However, Sp1 was the only transcription factor that was common to the results generated by the PROMO database and the RNA-Seq data. In a previous study, Chae et al demonstrated that a treatment that included ring-Sp1 decoy oligonucleotides could reduce the expression levels of Sp1 and thereby inhibit the expression levels of TGF-β1 and Fn in normal rat kidney fibroblasts (NRK-49F cells), thus indicating that Sp1 might promote the progression of RIF 18. Another *in vitro* study showed that the knockdown of Sp1 in mesangial cells suppressed the transcriptional activity of follistatin, an anti-fibrotic protein, thus suggesting that Sp1 might exert an anti-RIF effect 19. These two studies indicated opposite roles for Sp1 in renal fibrosis potentially due to differences in regulation of pathways in different microenvironments and in different cells. However, whether Sp1 participates in RIF by regulating the expression of Gm26669 has yet to be determined.

In the present study, we aimed to investigate the specific effect of Sp1 in RIF, explore the mechanisms underlying the regulatory effects of Sp1 on Gm26669, and clarify the role of the Sp1/Gm26669 axis in the anti-RIF effect of NCTD.

## Materials and methods

### Animal model

Healthy male C57BL/6J mice (8 weeks-of-age) were purchased from Hunan Slack King Experimental Animal Company (Changsha, China). The mice were placed in separate cages in the laboratory of the Experimental Animal Center of the Second Xiangya Hospital of Central South University. The mice experienced a 12-h/12-h light-dark cycle and had access to drinking water and standard laboratory food *ad libitum*. To construct the UUO model, mice were anesthetized by an intraperitoneal injection of pentobarbital (50 mg/kg) and the kidneys were separated and exposed layer by layer. The ureters were ligated with 4-0silk sutures at two points; we then made an incision between the two sutures and then closed the animal layer by layer. In the sham group, we only released the ureter without ligation. Mice in the NCTD-treated group were given a daily intraperitoneal injection of 0.075 mg/kg of NCTD (Sigma-Aldrich, USA) for 7 days; this treatment commenced on the first day of UUO surgery and was continued until the mice were sacrificed 16. The mice that did not receive NCTD treatment were given a corresponding volume of sterile saline *via* intraperitoneal injection. All mice were sacrificed 7 days after kidney surgery, and kidney tissue samples from each group were collected for further analysis. All experiments were approved by the Animal Ethnics Committee of the Second Xiangya Hospital.

### Intrarenal lentivirus delivery

We delivered Gm26669 shRNA or Gm26669 overexpression lentivirus (LV-Gm26669) directly into the kidneys of experimental mice by intraparenchymal injection 7 days before establishing the UUO model. The intraparenchymal delivery of lentivirus was performed using a technique that was described previously 20. In brief, mice were anesthetized with pentobarbital (60 mg/kg). After skin preparation, a 1 cm longitudinal incision was made on the left flank. The left renal pedicle was then temporarily blocked with a non-traumatic vascular clip and the needle was inserted into the lower pole of the kidney in a position that was parallel to its long axis. When the needle tip was close to the edge of the upper pole of the kidney, the injection was initiated and the needle was simultaneously withdrawn. Once the medicine had been completely injected, the needle was stopped for 15 s to prevent leakage, and then removed completely. The abdomen was then sutured layer by layer, disinfected, and the mice were kept warm until they came round from the anesthesia.

### Histomorphology and immunohistochemical (IHC) staining

Fresh kidney tissue was embedded in paraffin, sectioned (2-3 µm thickness), and dried in an oven at 60°C for 120 min prior to use. Following deparaffinization and hydration, the sections were stained with hematoxylin-eosin (HE) and a light microscope was used to visualize the morphology of the renal tissue. For IHC staining, sections were placed in citrate buffer (10 mM citric acid, pH 6.0) and heated (high heat for 8min, medium heat for 20min) in a microwave oven for antigen retrieval. Next, 3% H_2_O_2_ was added dropwise to block endogenous peroxidase activity. Each section was then incubated overnight at 4°C with an optimized concentration of Sp1 antibody (Abcam, 1:400), CoL-I (Affinity, 1:150), and Fn antibody (Abcam, 1:200). For the negative control, we used non-immune IgG instead of the primary antibody. The next morning, sections were washed and then incubated for 1 h at room temperature with HRP-labeled biotinylated goat anti-rabbit IgG. Positive antibody binding was subsequently detected with a diaminobenzidine (DAB) kit. Finally, representative images were acquired at a magnification of 400×.

### Cell culture

The mouse proximal tubular epithelial cell line (BUMPT) was gifted by Professor Dong Zheng from the Nephrology Institute of the Second Xiangya Hospital of Central South University. These cells were cultured in an incubator at 37°C and with an atmosphere containing 5% CO_2_. The cellular model of fibrosis was created by adding 5 ng/ml of TGF-β1 to BUMPT cells and incubating for 24 h 21, 22. The intervention concentration of NCTD was 5 µg/ml. NCTD-treated BUMPT cells were incubated with TGF-β1 for 24 h 23. Cells were then collected for western blotting and quantitative real-time PCR analysis.

### Extraction of the nuclear and cytoplasmic proteins

Proteins were extracted with a Nuclear and Cytoplasmic Protein Extraction Kit (Beyotime Institute of Biotechnology, China) in accordance with the manufacturer's instructions. Adherent cells were scraped and mixed with PMSF and cytoplasmic protein extraction reagent. Then, the cell lysates were vortexed (5 s) and centrifuged (12000-16000 g for 5 min at 4℃); the supernatant was then taken the source of cytoplasmic proteins. Finally, the remaining precipitate was mixed with PMSF and nucleoprotein extraction reagent, vortexed, and centrifuged. The final supernatant was taken as the nuclear protein.

### Cell transfection

Lipofectamine 2000 reagent (Invitrogen, USA) was used to transfect Sp1 siRNA, scramble siRNA (Scr siRNA), and a Gm26669 overexpression lentivirus (LV-Gm26669), or negative control lentivirus (LV-NC), in accordance with the manufacturer's instructions. After 6 h of transfection, the cells were cultured with 5 ng/ml of TGF-β1 for a further 24 h.

### Western blotting

We used the BCA Protein Detection Kit (Pierce Biotechnology, USA) to determine the concentration of each protein sample. Western blotting was then carried out in accordance with standard procedures. Membranes were incubated overnight withspecific primary antibodies, as follows: anti-Fn antibody (Abcam, 1:1500), anti-CoL-1 antibody (Affinity, 1:1000), anti-Sp1 antibody (Abcam, 1:5000), and anti-β-actin antibody (Proteintech, 1:3000). The next morning, the membranes were washed and incubated for 1 h with peroxidase-conjugated goat anti-rabbit immunoglobulin G (secondary antibody). Membrane binding to the antibodies was detected with the enhanced chemiluminescence system. Finally, band intensity was evaluated by Image J software (National Institutes of Health, Bethesda, MD).

### Quantitative real-time PCR (qRT-PCR)

Cells were washed three times with sterile enzyme-free PBS and then mixed with 1 ml of Trizol (Invitrogen) for lysis and total RNA extraction. Next, we used the PrimeScript RT reagent Kit with gDNA Eraser (Takara) to synthesize first-strand cDNA. cDNA samples were then amplified using a LightCycler 480 PCR System and a SYBR Premix Ex TaqⅡ Kit (Takara). Following amplification, we evaluated the amplification and dissolution curves and then determined the CT value for each sample. β-actin was used as an internal reference. The primer sequences used for PCR amplification are shown in Table 1.

### Chromatin immunoprecipitation (CHIP) assays

Cells were first incubated with 1.1% formaldehyde at room temperature for 10 min. Then, cell lysate was collected and disrupted ultrasonically (ultrasound for 6 s, rest for 4 s for a total period of 30 s; this was repeated 3 times at 1 min intervals at 50% power). Next, the cell lysate was incubated with Sp1 antibody at 4℃ overnight to combine with the target protein-DNA complex. After de-crosslinking and DNA purification, enriched DNA fragments were obtained, and then analyzed by PCR.

### Immunofluorescence (IF) staining

Kidney tissues were blocked with 3% H_2_O_2_ and then incubated overnight with anti-Sp1 antibody (Abcam, 1:500) at 4°C. The following morning, the sections were washed and then incubated with secondary antibody at room temperature for 1h in the dark (Proteintech, 1:100). Sections were then stained with DAPI, mounted with an anti-fluorescence quencher, and then observed and photographed under a fluorescence microscope.

Cells were by fixed by 4% paraformaldehyde and blocked with 10% goat serum at room temperature for 1 h. Then, the cells were incubated with anti-Sp1 antibody (Abcam, 1:500) at 4°C overnight. Unlike the IF staining of tissues, we used Hoechst (not DAPI) to stain the nucleus and the cells were finally observed and imaged using a confocal laser scanning microscope (Zeiss LSM 780, Germany).

### Statistical analysis

Data were analyzed using SPSS 20.0 software and visualized by GraphPad Prism Version 7.0. All data were expressed as mean ± standard error of the mean (SEM). Correlations between the two variables were evaluated using Pearson's correlation coefficient. Statistical differences were analyzed using the student's t-test for two groups and by one-way analysis of variance ANOVA for multiple groups. Bonferroni correction was applied for multiple comparisons to correct P values. P < 0.05 was regarded as statistically significant.

## Results

### The expression of Sp1 in the kidneys of UUO mice and BUMPT cells exposed to TGF-β1

**Figure 1:**
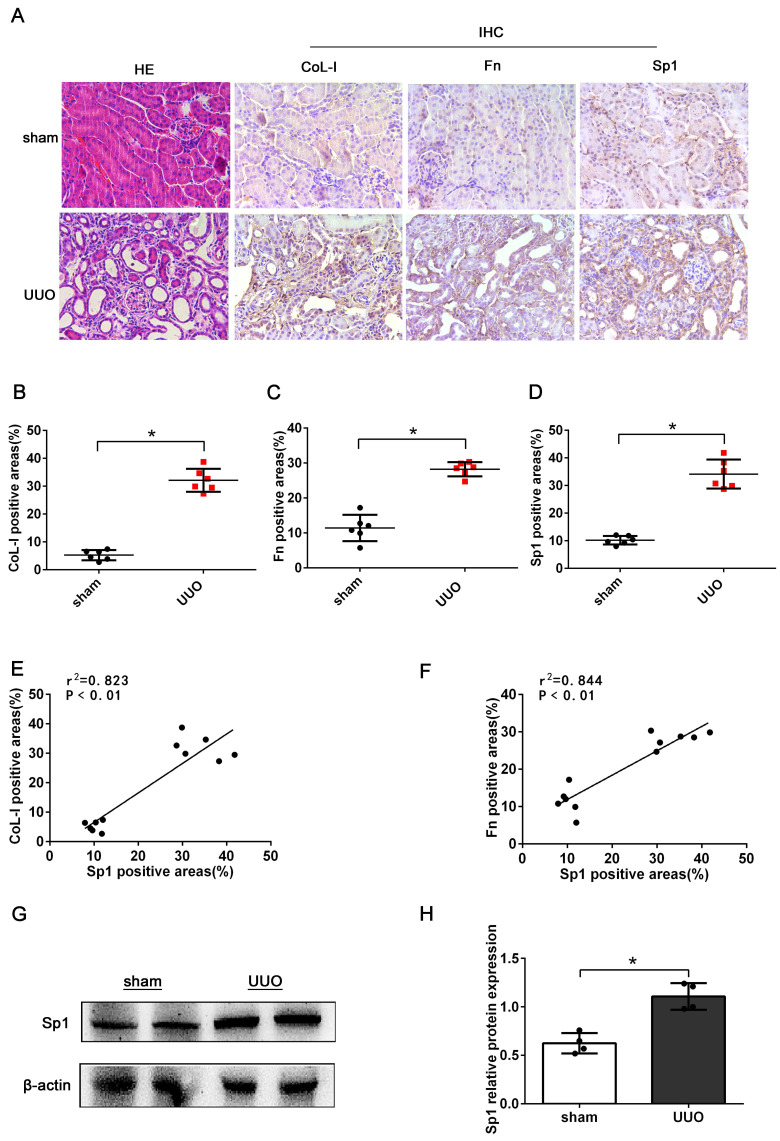
** The expression levels of Sp1 in UUO kidneys.** (A) HE staining of the kidneys of UUO mice and the IHC analysis of CoL-1, Fn, and Sp1 in renal tissues (400×). (B-D) Areas showing positive immunostaining for CoL-1, Fn, and Sp1 in mice kidneys. Data are presented as mean ±SEM (n=6). (E-F) Correlation analysis of CoL-I and Fn expression levels with Sp1, as determined by the IHC analysis of mice kidneys (n=6). (G-H) Western blotting and densitometric analyses of Sp1 expression in the kidneys of UUO mice; *P<0.05, data are presented as the mean ±SEM (n=4).

First, we investigated the expression of Sp1 in the kidneys of UUO mice (7 days after model establishment). HE staining revealed dilated tubules, the loss of brush border, tubular atrophy, and inflammatory cell infiltration in UUO mice, thus suggesting that the model of UUO had been established successfully (Fig. 1A). IHC staining showed increased levels of CoL-I, Fn, and Sp1 in the kidneys of UUO mice, compared with the sham group; and revealed that Sp1 was mainly localized to the nucleus of renal tubular epithelial cells (Fig. 1A-D). In addition, linear regression showed that the expression levels of CoL-1 and Fn were positively correlated with the expression levels of Sp1 (Fig. 1E, F). Western blotting results also confirmed that the protein levels of Sp1 were elevated in the kidneys of UUO mice (Fig. 1G, H).

**Figure 2:**
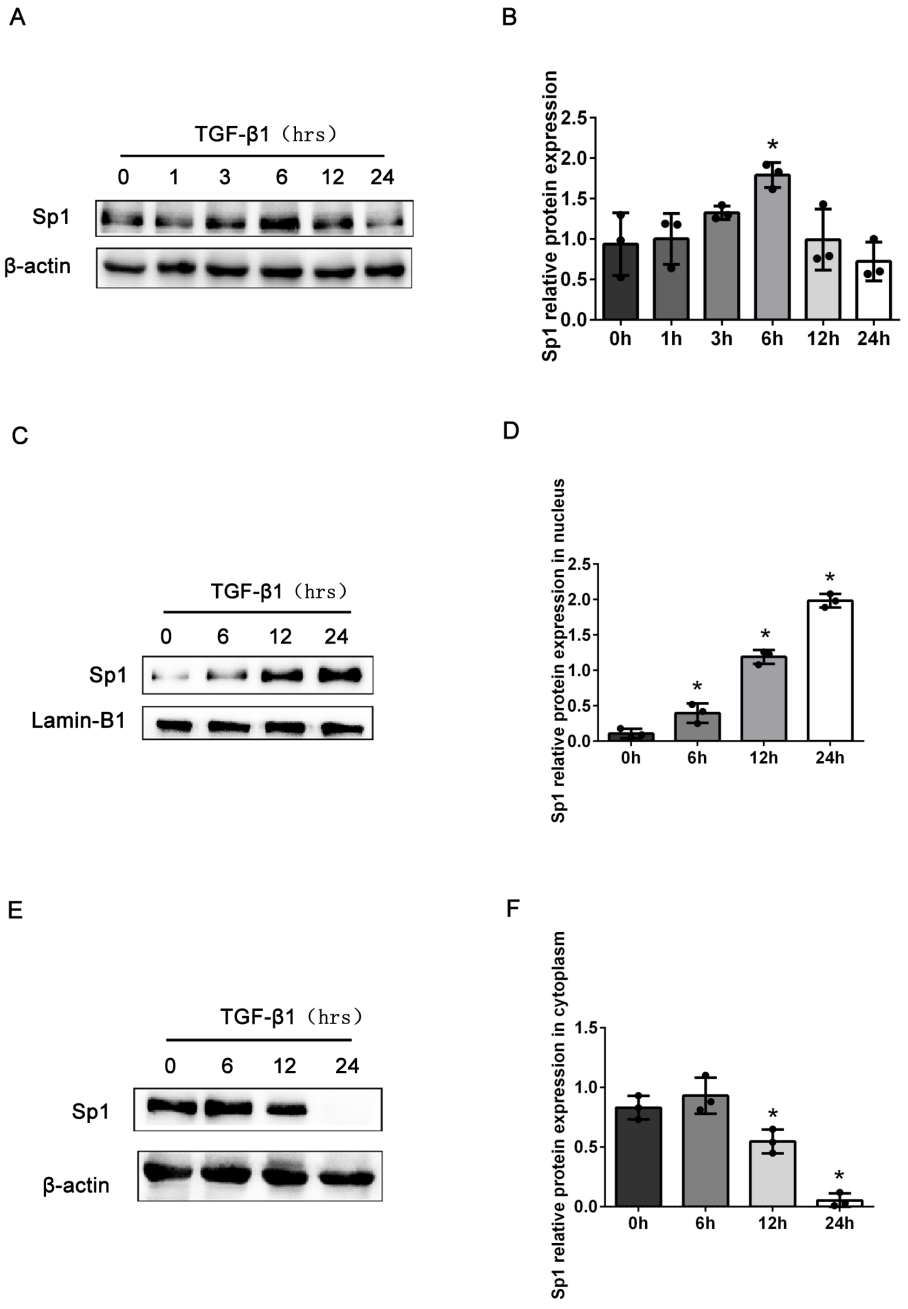
** The expression levels of Sp1 in BUMPT cells exposed to TGF-β1.** (A-B) Western blotting and densitometric analyses of Sp1 expression in whole BUMPT cells after stimulation with TGF-β1(5 ng/ml). (C-D) Western blotting and densitometric analyses of Sp1 expression in the nuclei of BUMPT cells after TGF-β1 stimulation. (E-F) Western blotting and densitometric analyses of Sp1 expression in the cytoplasm of BUMPT cells after TGF-β1 stimulation. *P<0.05, ^#^P<0.05, ^&^P<0.05; data are presented as the mean ±SEM (n=3).

Next, we detected the expression levels of Sp1 in BUMPT cells stimulated with TGF-β1 at different time points. Compared with the control group, the total expression levels of Sp1 protein were markedly elevated in BUMPT cells after stimulation with TGF-β1 for 6 h, and then decreased with the prolong of TGF-β1 stimulation time (Fig. 2A, B). However, previous studies have demonstrated obvious cellular fibrosis when cells were exposed to TGF-β1 for 24 h, which were different from the expression trend of Sp1 21. Since Sp1 was mainly expressed in the nuclei of renal tubular epithelial cells, we next detected the expression levels of Sp1 in the nuclei and cytoplasm. Western blotting showed that the expression levels of Sp1 protein followed a gradual time-dependent increase in the nuclei but were reduced in the cytoplasm (Fig. 2C-F). These data suggested that Sp1 underwent nuclear translocation in BUMPT cells following TGF-β1 stimulation.

### The knockdown of Sp1 inhibited the expressions of ECM-related genes induced by TGF-β1

**Figure 3:**
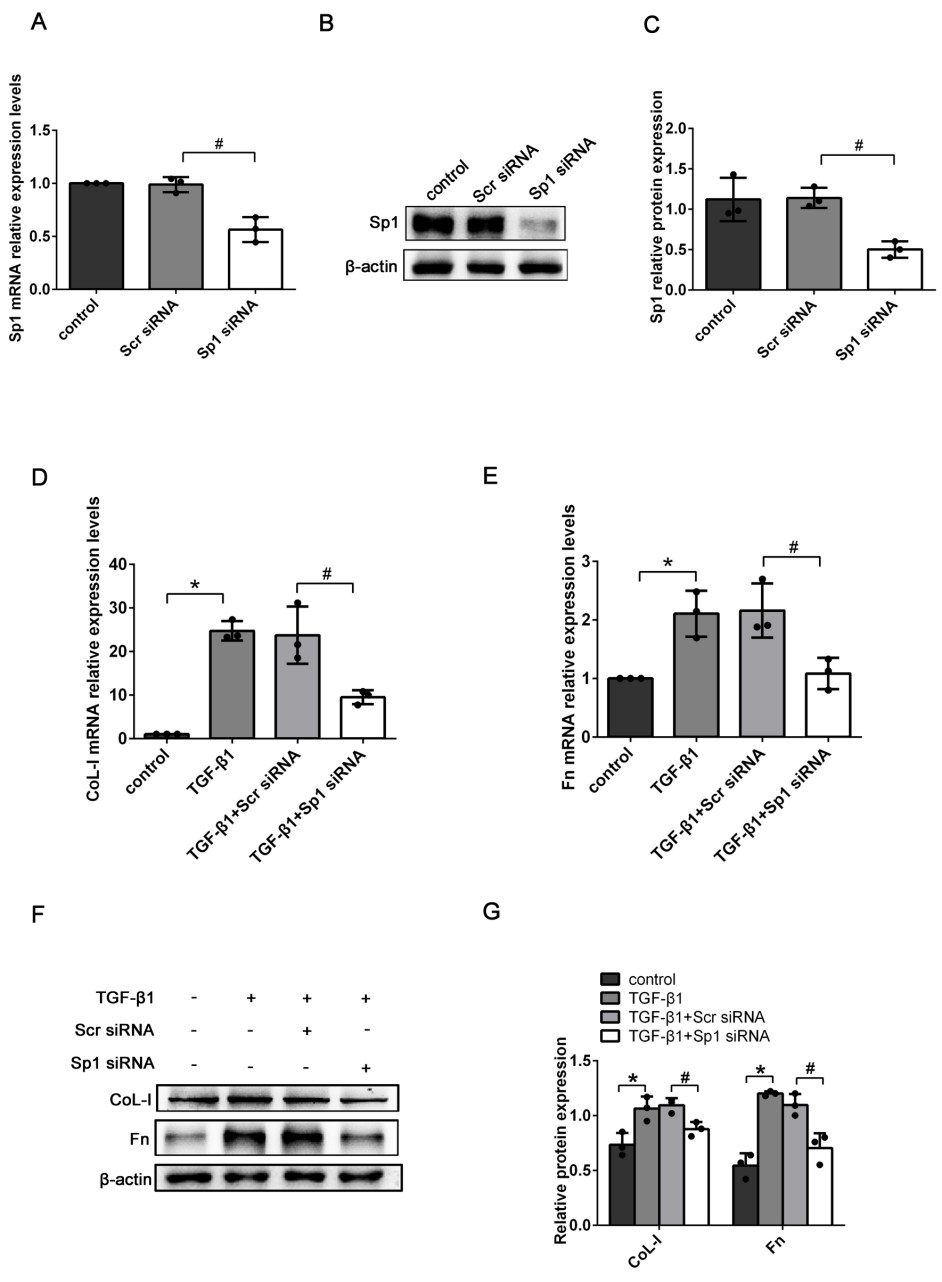
** The knockdown of Sp1 inhibited TGF-β1-induced ECM expression.** (A-C) qRT-PCR, western blotting and densitometric analyses of Sp1 expression in BUMPT cells following the transfection of Sp1 siRNA. (D-E) qRT-PCR analysis of the mRNA expression levels of CoL-I and Fn in TGF-β1-stimulated BUMPT cells following transfection with Sp1 siRNA. (F-G) Western blotting and densitometric analyses of CoL-I and Fn expression level in TGF-β1-stimulated BUMPT cells following Sp1 siRNA transfection. *P<0.05, ^#^P<0.05; data are presented as the mean ±SEM (n=3).

To determine the exact role of Sp1 in RIF, the expression levels of ECM-related genes (CoL-I and Fn) were detected in Sp1 siRNA-transfected BUMPT cells. qRT-PCR and western blotting showed that compared with scramble siRNA (scr siRNA) -transfected cells, the expression levels of Sp1 mRNA and protein were greatly reduced in Sp1 siRNA-transfected cells, thus suggesting that Sp1 siRNA had been successfully transfected into BUMPT cells (Fig. 3A-C). In addition, we found that the silencing of Sp1 inhibited the increase of CoL-I and Fn mRNA and protein expressions in BUMPT cells exposed to TGF-β1 over a 24 h period (Fig. 3D-G).

### Sp1 promoted the expression of ECM-related genes by targeting Gm26669

**Figure 4:**
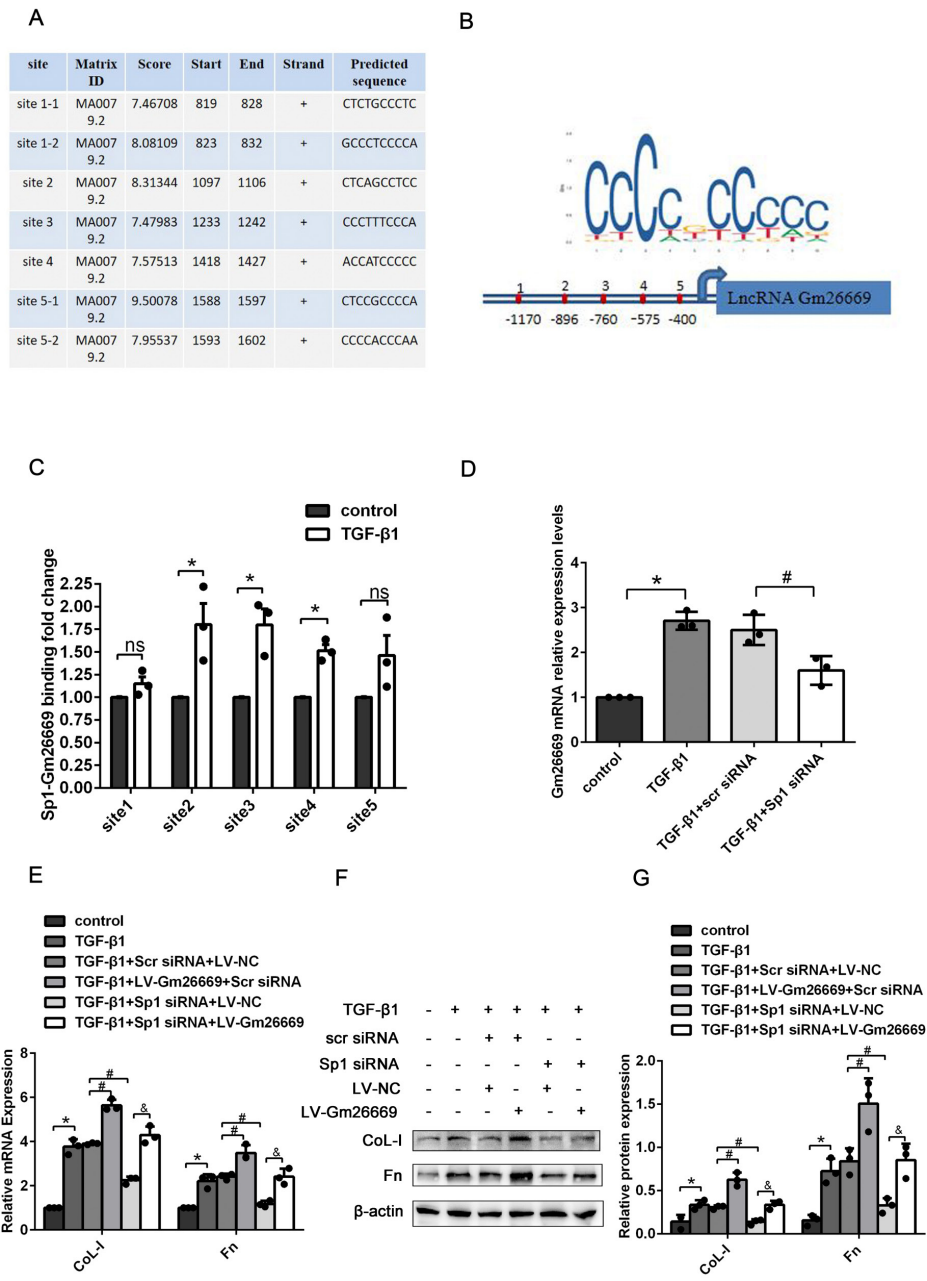
** Sp1 regulated the expression of Gm26669 and mediated the expression of ECM-related genes by acting on the Gm26669 promoter.** (A-B) JASPAR identified five binding sites for Sp1 in the Gm26669 promoter region. (C) CHIP experiments were performed to investigate the changes in binding between Sp1 and the Gm26669 promoter in BUMPT cells when stimulated by TGF-β1. (D) qRT-PCR analysis of Gm26669 expression levels in TGF-β1-stimulated BUMPT cells following Sp1 siRNA transfection. (E-G) qRT-PCR, western blotting and densitometric analyses of CoL-I and Fn expression levels in TGF-β1-stimulated BUMPT cells following transfection with Sp1 siRNA and LV-Gm26669. *P<0.05, ^#^P<0.05, ^&^P<0.05, ^^^P<0.05; data are presented as the mean ±SEM (n=3).

Furthermore, we used the transcription factor prediction software JASPAR to identify potential binding sites on the Gm26669 promoter for Sp1. Our analysis identified seven binding sites that exhibited high scores between Sp1 and the Gm26669 promoter. After merging two overlapping sites, we selected five binding sites for further analysis (Fig. 4A, B). CHIP assays revealed that Sp1 could bind directly to the Gm26669 promoter and that TGF-β1 stimulation significantly increased Sp1 enrichment at the second, third, and fourth binding sites of Gm26669 promoter, compared with the control group (Fig. 4C). Thus, we speculated that Sp1 might be an upstream regulator of Gm26669. To confirm this hypothesis, we next determined the expression level of Gm26669 in cells transfected with Sp1 siRNA. qRT-PCR showed that the silencing of Sp1 inhibited the expression of Gm26669 in cells exposed to TGF-β1, thus indicating that Sp1 could regulate the expression of Gm26669 (Fig. 4D). In addition, qRT-PCR and western blotting showed that the overexpression of Gm26669 was able to rescue the inhibitory effects of Sp1 silencing on the mRNA and protein expression levels of CoL-I and Fn (Fig. 4E-G).

### NCTD prevented RIF by inhibiting the expression of Gm26669 in the kidneys of UUO mice and BUMPT cells exposed to TGF-β1

**Figure 5:**
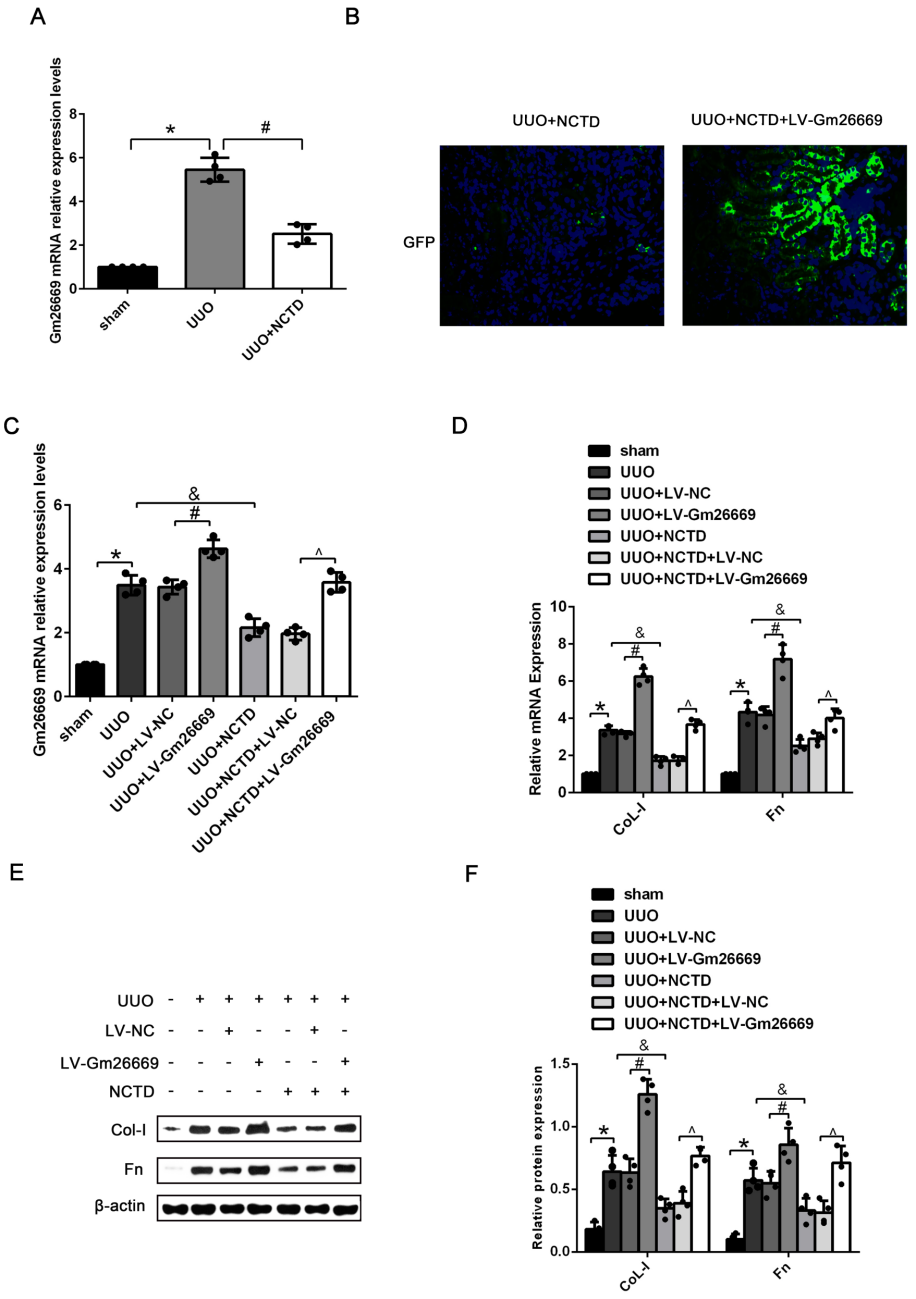
** The effects of NCTD on the deposition of ECM in the kidneys of UUO mice following the injection of LV-Gm26669.** (A) qRT-PCR analysis of the expression levels of Gm26669 in the kidneys of UUO mice following NCTD treatment. (B) GFP immunofluorescence in the kidneys of UUO mice injected with LV-Gm26669 and treated with NCTD (400×). (C) qRT-PCR analyses of Gm26669 expression levels in the kidneys of UUO mice injected with LV-Gm26669 and treated with NCTD. (D-F) qRT-PCR, western blotting and densitometric analyses of the expression levels of CoL-I and Fn in the kidneys of UUO mice injected with LV-Gm26669 and treated with NCTD. *P<0.05, ^#^P<0.05, ^&^P<0.05, ^P<0.05, data are presented as the mean ±SEM (n=4).

Our present results showed that NCTD treatment significantly reduced the expression of Gm26669 in the kidneys of UUO mice (Fig. 5A). IF analysis and qRT-PCR showed that the Gm26669 overexpression lentivirus (LV-Gm26669) had been successfully injected into the renal tubules of mice (Fig. 5B, C). qRT-PCR and western blotting further revealed that the overexpression of Gm26669 significantly increased the mRNA and protein expression levels of CoL-I and Fn in the kidneys of UUO mice; NCTD treatment greatly inhibited the expression levels of CoL-I and Fn. Furthermore, the overexpression of Gm26669 weakened the inhibitory effect of NCTD on the mRNA and protein expression levels of CoL-I and Fn (Fig. 5D-F). Similarly, the efficacy of the Gm26669 shRNA *in situ* kidney injection was also investigated by IF staining and qRT-PCR (Supplementary Fig. 1A, B). The injection of Gm26669 shRNA led to a significant inhibition of the mRNA and protein expression of CoL-I and Fn in the kidneys of UUO mice. However, compared with NCTD treatment alone, the combined treatment of Gm26669 shRNA transfection and NCTD showed no further reduction in the expression levels of CoL-I and Fn (Supplementary Fig. 1C-E).

**Figure 6:**
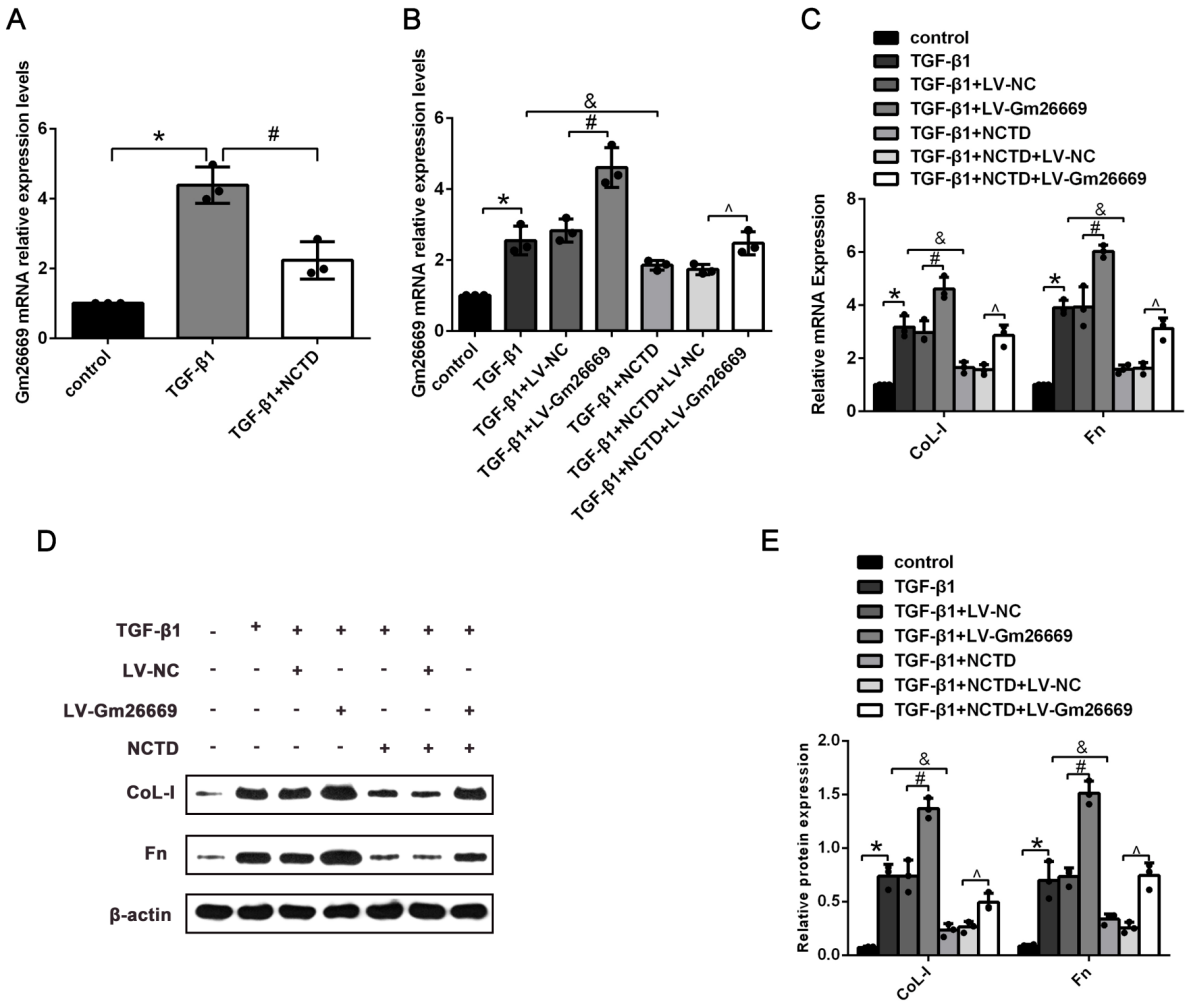
** The effects of NCTD on ECM deposition in BUMPT cells with LV-Gm26669 transfection.** (A) qRT-PCR analysis of Gm26669 expression in BUMPT cells following TGF-β1 stimulation and NCTD treatment. (B) qRT-PCR analyses of Gm26669 expression in TGF-β1-stimulated BUMPT cells following transfection with LV-Gm26669 and NCTD treatment. (C-E) Western blotting and densitometric analyses of CoL-I and Fn expression levels in TGF-β1-stimulated BUMPT cells following transfection with LV-Gm26669 and NCTD treatment. *P<0.05, ^#^P<0.05, ^&^P<0.05, ^^^P<0.05; data are presented as the mean ± SEM (n=3).

qRT-PCR results showed that the expression levels of Gm26669 were increased in LV-Gm26669-transfected cells and reduced by NCTD treatment (Fig. 6A-B). In addition, qRT-PCR and western blotting showed that the overexpression of Gm26669 greatly weakened the inhibitory effect of NCTD on the mRNA and protein expression levels of CoL-I and Fn in TGF-β1-stimulated BUMPT cells (Fig. 6C-E). In contrast, the expression levels of Gm26669 were reduced in Gm26669 shRNA-transfected cells but were not reduced further in cells when combined with Gm26669 shRNA transfection and NCTD treatment. Moreover, the inhibitory effects of NCTD on the expression of CoL-I and Fn were not further enhanced by the application of Gm26669 shRNA (Supplementary Fig. 2A-D).

### NCTD inhibited the nuclear translocation of Sp1 in the kidneys of UUO mice and BUMPT cells exposed to TGF-β1

**Figure 7:**
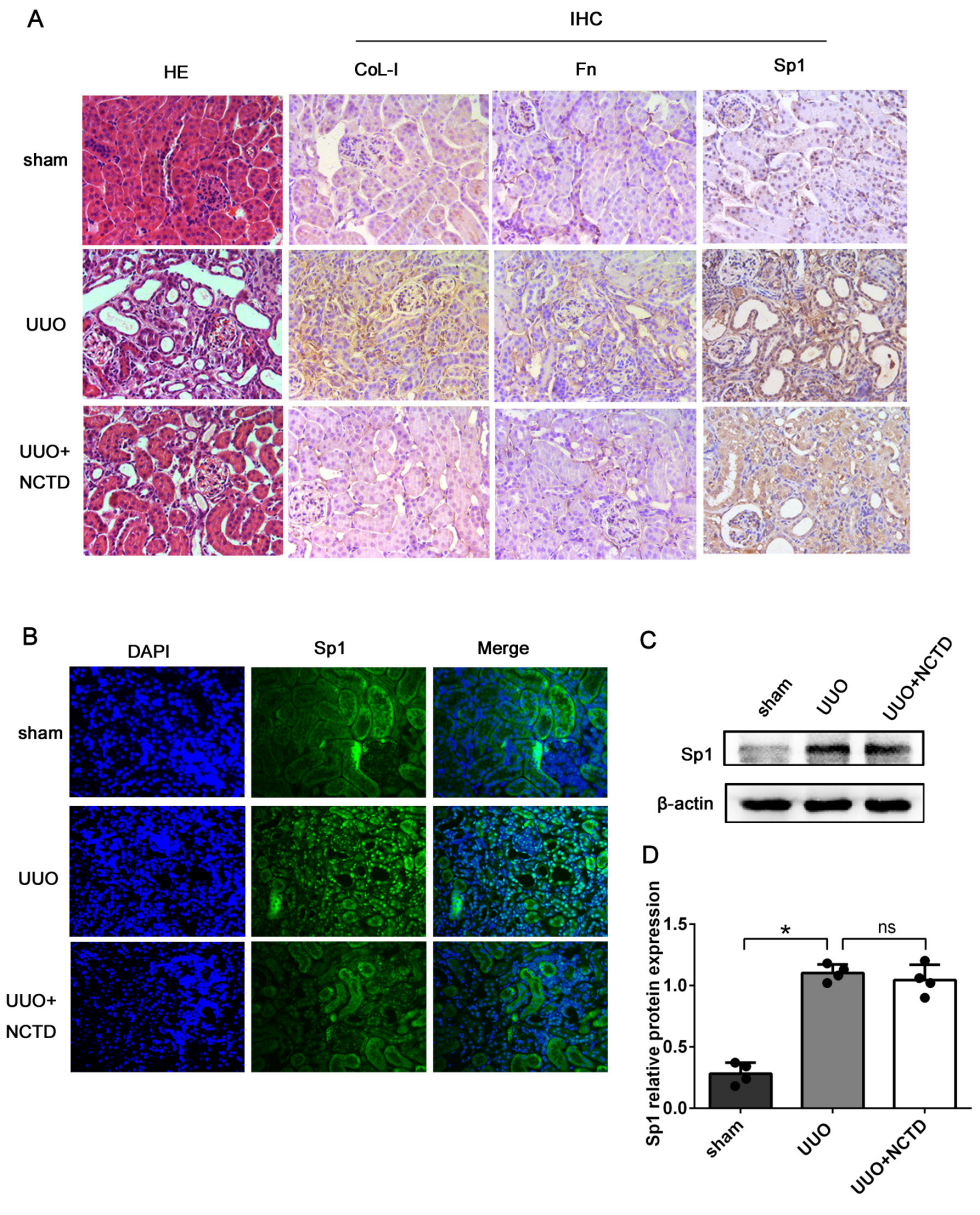
** NCTD inhibited the nuclear translocation of Sp1 in the kidneys of UUO mice.** (A) HE staining of mouse kidneys and the IHC staining of CoL-1, Fn, and Sp1 in the kidneys of UUO mice following NCTD treatment (400×). (B) IF analysis of Sp1 in the kidneys of UUO mice following NCTD treatment (400×). (C-D) Western blotting and densitometric analyses of Sp1 expression in the kidneys of UUO mice following NCTD treatment. *P<0.05, ^#^P<0.05; data are presented as the mean ± SEM (n=4).

Next, we investigated the regulatory role of NCTD on the expression and nuclear translocation of Sp1. HE staining revealed that the degree of renal tubule dilatation and atrophy were mitigated by NCTD intervention. Furthermore, the expression levels of CoL-I and Fn in the tubulointerstitium of UUO mice were decreased following NCTD intervention when compared with the UUO group, thus further confirming the protective effects of NCTD (Fig. 7A). IHC and IF staining showed that the expression levels of Sp1 in the nuclei of the renal tubular epithelial were significantly elevated in UUO mice; in contrast, the application of NCTD inhibited the nuclear expression of Sp1 (Fig. 7A, B). Nevertheless, western blotting analysis showed that NCTD had no significant effect on the expression levels of Sp1 protein in samples of whole kidney tissue (Fig. 7C, D).

**Figure 8:**
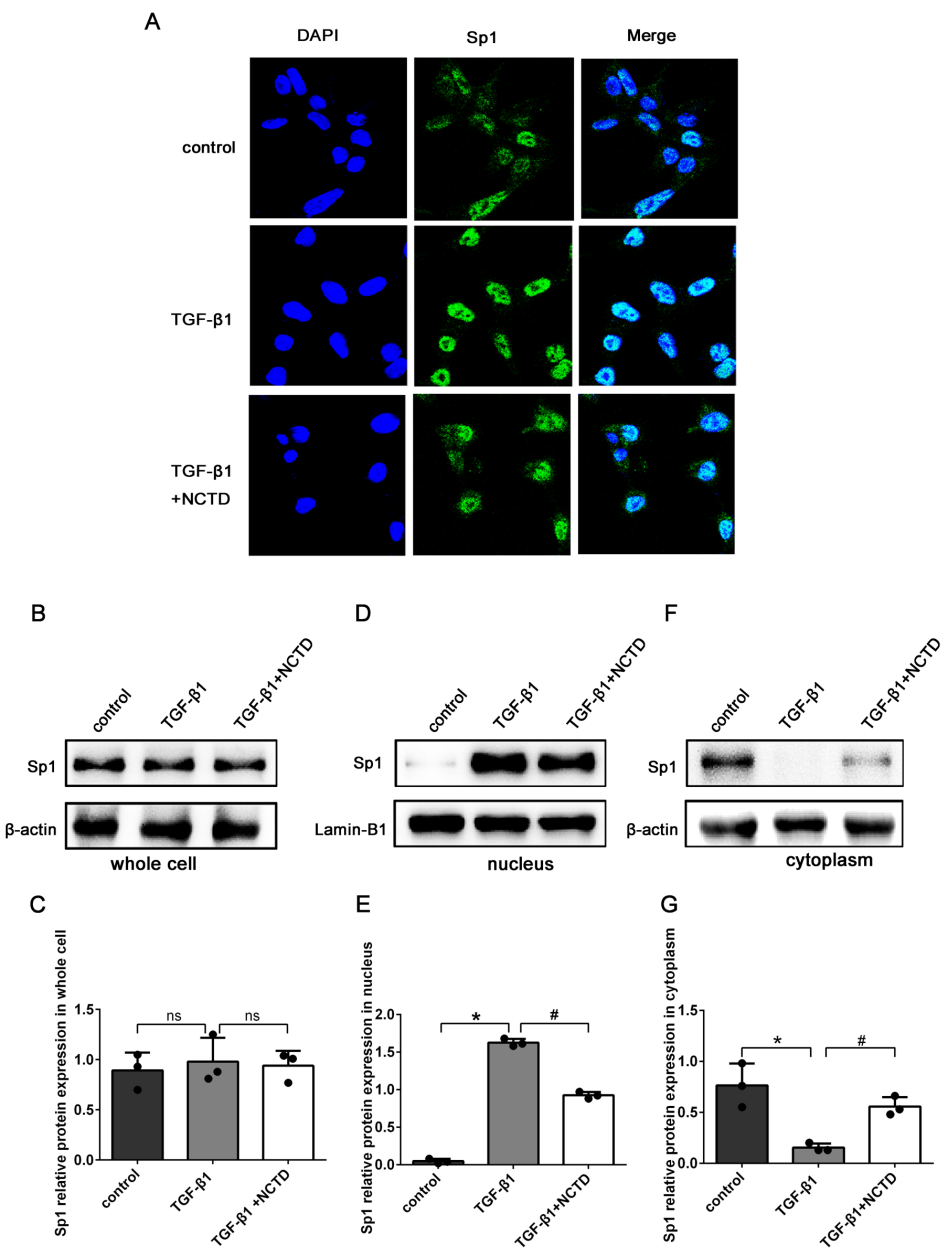
** NCTD inhibited the nuclear translocation of Sp1 in BUMPT cells when stimulated by TGF-β1.** (A) IF analysis of Sp1 in BUMPT cells following TGF-β1 stimulation and NCTD treatment (400×). (B-G) Western blotting and densitometric analyses of Sp1 expression levels in whole cells, nuclei, and the cytoplasm following TGF-β1 stimulation and NCTD treatment. *P<0.05, ^#^P<0.05; data are presented as the mean ± SEM (n=3).

In addition, IF staining showed that compared with the control group, the expression levels of Sp1 in the nuclei of BUMPT cells were significantly enhanced in cells stimulated with TGF-β1. The application of NCTD significantly reduced the nuclear translocation of Sp1 (Fig. 8A). Western blotting confirmed that the total protein expression levels of Sp1 remained unchanged after NCTD treatment (Fig.8B-C). However, the application of NCTD reduced the expression of Sp1 in the nuclei and increased the expression of Sp1 in the cytoplasm (Fig. 8D-G).

## Discussion

RIF is the common pathological process underlying various CKDs 24. The activation and migration of myofibroblasts, inflammation, as well as the deposition of ECM, are all known to be involved in the pathogenesis of RIF. Various signaling pathways such as TGF-β1/Smad, Wnt/β-catenin, PI3K/Akt, PKC/ERK, and Notch, have been reported to play an important role in RIF 25, 26. However, the pathogenesis of RIF has yet to be fully elucidated.

In recent years, a growing number of studies have demonstrated that lncRNAs participate in the development of RIF by playing a regulatory role in the pathogenesis of inflammation, the activation and proliferation of fibroblast, and the accumulation of ECM 27. Although we previously confirmed that Gm26669 promoted RIF both *in vivo* and *in vitro* (unpublished data), the specific mechanism involved in regulating Gm26669 expression was unclear. Combined with the results of bioinformatics analysis and our previous RNA-Seq, we found that Sp1 was the only potential transcription factor involved in the regulation of Gm26669 by NCTD.

Sp1 is a member of the Sp/Kruppel-like zinc finger transcription factor family, which can bind to the GC box via its zinc finger structure to play an important biological role in cell proliferation, apoptosis, and differentiation 28. At present, the precise role of Sp1 in RIF remains controversial 18, 19.

In the present study, we found that the expression levels of Sp1 increased in the kidneys of UUO mice and were positively correlated with the expression levels of both CoL-I and Fn. In addition, Sp1 was mainly localized to the nuclei of the renal tubular epithelial cells. From a functional point-of-view, the silencing of Sp1 markedly inhibited the expression levels of CoL-I and Fn, thus suggesting that Sp1 plays a positive role in RIF. However, total Sp1 protein may not participate in the progression of RIF as indicated by the fact that the total expression levels of Sp1 were not significantly increased in BUMPT cells exposed to TGF-β1 over a 24 h period. Further studies found that the expression levels of Sp1 were gradually elevated in the nucleus but fell gradually in the cytoplasm as the TGF-β1 stimulation time increased. Taken together, our results suggested that increased nucleic translocation of Sp1 promotes the progression of RIF.

Sp1 contains three Cys2-His2 zinc finger structures that act as nuclear localization signals and can be transported to the nucleus by typical nuclear localization pathways mediated by endotransport protein α and endotransport protein β, thereby combining with GC base-rich gene promoter sequences to regulate the expression of target genes 29, 30. Under high glucose conditions, the overexpression of Sp1 bound to the promoter of the lncRNA MALAT1 to activate MALAT1 transcription and aggravate diabetic retinopathy 31. In this study, we used JASPAR prediction software to acquire evidence to suggest that Sp1 might bind to the promoter of Gm26669; this was further confirmed by our CHIP assays. We also found that the ability of Sp1 to bind to the Gm26669 promoter was enhanced by TGF-β1 stimulation. At the functional level, the upregulation of Gm26669 induced by TGF-β1 was inhibited by Sp1 siRNA. From a mechanical viewpoint, the overexpression of Gm26669 reversed the inhibitory effect of Sp1 siRNA on the expression levels of CoL-I and Fn. Taken together, these results indicated that the Sp1 transcription factor might promote RIF by binding to the promoter of Gm26669 and increasing the expression levels of Gm26669.

By targeting the transcription of non-coding RNA, it might be possible to influence the progression of fibrotic diseases. For example, liquiritigenin can inhibit liver fibrosis by inhibiting the miR-181b/PTEN pathway 32. Moreover, studies have shown that antisense oligonucleotide therapy targeted to lncMGC can reduce ECM deposition in the glomeruli of diabetic mice 33, 34. Our previous studies demonstrated that NCTD could successfully alleviate the degree of RIF 8-11. By applying RNA-seq, we found that NCTD reduced the expression levels of Gm26669 16. In the present study, we demonstrated that the overexpression of Gm26669 could weaken the inhibitory effect of NCTD on ECM deposition. Furthermore, the knockdown of Gm26669 did not lead to a further enhancement in the inhibitory role of NCTD on the accumulation of ECM, thus suggesting that Gm26669 plays a critical role in the anti-fibrotic effects of NCTD.

A number of drugs (such as first-line cytotoxic drugs, non-steroidal anti-inflammatory drugs, cyclooxygenase-2 inhibitors, metformin, and anti-tumor drugs) have been shown to reduce the expression levels of Sp1 or interfere with the binding of Sp1 to DNA to inhibit the transcription of target genes 35, 36. In this study, we found that the administration of NCTD inhibited the nuclear translocation of Sp1 in the renal tubular epithelial cell of UUO mice. Similarly, the application of NCTD reduced the nuclear translocation of Sp1 in TGF-β1-stimulated BUMPT cells. However, NCTD had no effect on the expression levels of total Sp1 protein in whole cells. Thus, we speculate that NCTD might inhibit the nuclear translocation of Sp1 to block the interaction between Sp1 and the promoter of Gm26669. However, the mechanisms responsible for how NCTD enters the cell to regulate the nuclear translocation of Sp1 deserves further exploration in the future.

## Conclusion

**Figure 9:**
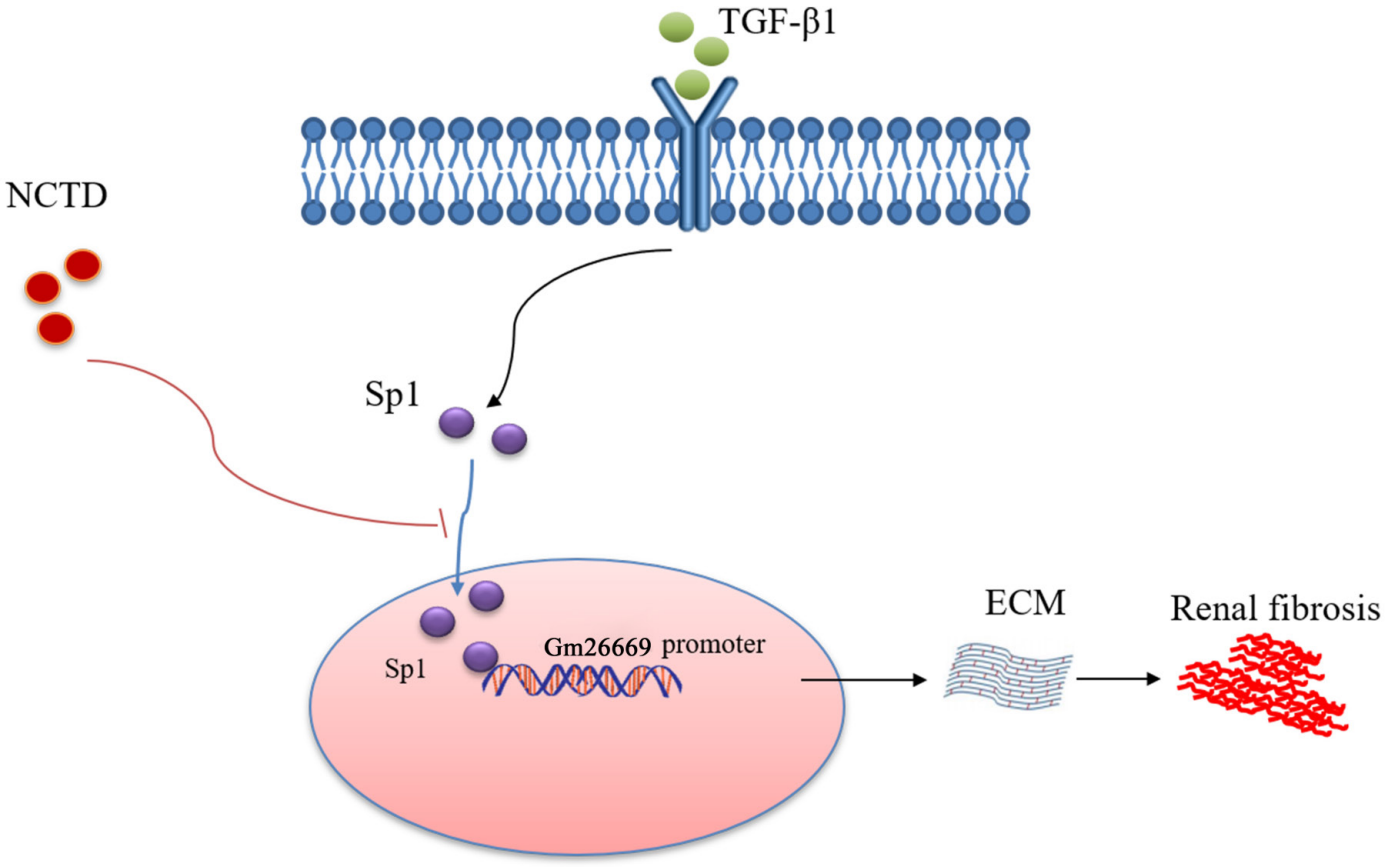
** Schematic representation of a working model that depicts how NCTD prevents renal interstitial fibrosis by targeting the Sp1/Gm26669 axis.** Following the stimulation of renal tubular epithelial cell by TGF-β1, Sp1 undergoes nuclear translocation and then combines with the promoter of the lncRNA Gm26669 to promote its transcription, thus leading to aggravation of renal interstitial fibrosis. NCTD reduces the expression of lncRNA Gm26669 by inhibiting the nuclear translocation of Sp1, thereby preventing renal interstitial fibrosis.

As summarized in Figure 9, we demonstrated that Sp1 promoted the progression of RIF by translocating into the nucleus to bind to the Gm26669 promoter and subsequently increase the transcription level of Gm26669. We also demonstrated that NCTD inhibited the nuclear translocation of Sp1 and prevented the binding of Sp1 to the promoter of Gm26669, thereby alleviating RIF. Our results provide a significant new insight into the pathogenesis of RIF and a theoretical basis for the application of NCTD in the clinic.

## Supplementary Material

Supplementary figures.

## Figures and Tables

**Table 1:** The primer sequences used for qRT-PCR.

Gene	Forward (5'-3')	Reverse (5'-3')
Gm26669	ACTTATGTCAAAACCTTCGTCCA	CTTTTACTTCCCAGCCCGAAC
Fn	GTGGCTGCCTTCAACTTCTC	TTGCAAACCTTCAATGGTCA
CoL-I	ACGTCCTGGTGAAGTTGGTC	TCCAGCAATACCCTGAGGTC
β-actin	AATCGTGCGTGACATCAAAGA	CCATACCCAAGAAGGAAGGC

## Abbreviations

RIF: renal interstitial fibrosis
NCTD: norcantharidin
Sp1: special protein 1
UUO: unilateral ureteral obstruction
CoL-I: collagen type I
Fn: fibronectin
CKD: chronic kidney disease
ECM: extracellular matrix
lncRNA: long non-coding RNA
HE: hematoxylin-eosin
BUMPT: a mouse proximal tubular epithelial cell line
Scr siRNA: scramble siRNA
LV-Gm26669: Gm26669-overexpressing lentivirus
LV-NC: negative control lentivirus
qRT-PCR: quantitative real-time PCR
CHIP: chromatin immunoprecipitation

## References

[1] Global regional, national burden of chronic kidney disease 1990-2017 (2020). a systematic analysis for the Global Burden of Disease Study 2017. Lancet (London, England).

[2] Humphreys BD (2018). Mechanisms of Renal Fibrosis. Annual review of physiology.

[3] Liu BC, Tang TT, Lv LL (2018). Renal tubule injury: a driving force toward chronic kidney disease. Kidney international.

[4] Weir MR, Lakkis JI, Jaar B (2018). Use of Renin-Angiotensin System Blockade in Advanced CKD: An NKF-KDOQI Controversies Report. American journal of kidney diseases: the official journal of the National Kidney Foundation.

[5] Zhang Z, Yang L, Hou J (2018). Promising positive liver targeting delivery system based on arabinogalactan-anchored polymeric micelles of norcantharidin. Artificial cells, nanomedicine, and biotechnology.

[6] Jiang S, Li M, Hu Y (2018). Multifunctional self-assembled micelles of galactosamine-hyaluronic acid-vitamin E succinate for targeting delivery of norcantharidin to hepatic carcinoma. Carbohydrate polymers.

[7] Xiao Z, Wen L, Zeng D (2020). Protein Phosphatase 2A Inhibiting β-Catenin Phosphorylation Contributes Critically to the Anti-renal Interstitial Fibrotic Effect of Norcantharidin. Inflammation.

[8] Liu FY, Li Y, Peng YM (2008). Norcantharidin ameliorates proteinuria, associated tubulointerstitial inflammation and fibrosis in protein overload nephropathy. American journal of nephrology.

[9] Li Y, Chen Q, Liu FY (2011). Norcantharidin inhibits the expression of extracellular matrix and TGF-β1 in HK-2 cells induced by high glucose independent of calcineurin signal pathway. Laboratory investigation; a journal of technical methods and pathology.

[10] Li Y, Ge Y, Liu FY (2012). Norcantharidin, a protective therapeutic agent in renal tubulointerstitial fibrosis. Molecular and cellular biochemistry.

[11] Li Y, Sun Y, Liu F (2013). Norcantharidin inhibits renal interstitial fibrosis by blocking the tubular epithelial-mesenchymal transition. PloS one.

[12] Luo HW, Yin DD, Xiao Z (2020). Anti-renal interstitial fibrosis effect of norcantharidin is exerted through inhibition of PP2Ac-mediated C-terminal phosphorylation of Smad3. Chemical biology & drug design.

[13] Peng PH, Chieh-Yu Lai J, Hsu KW (2020). Hypoxia-induced lncRNA RP11-390F4.3 promotes epithelial-mesenchymal transition (EMT) and metastasis through upregulating EMT regulators. Cancer letters.

[14] Lellahi SM, Rosenlund IA, Hedberg A (2018). The long noncoding RNA NEAT1 and nuclear paraspeckles are up-regulated by the transcription factor HSF1 in the heat shock response. The Journal of biological chemistry.

[15] Han R, Hu S, Qin W (2019). Upregulated long noncoding RNA LOC105375913 induces tubulointerstitial fibrosis in focal segmental glomerulosclerosis. Scientific reports.

[16] Xiao H, Liao Y, Tang C (2019). RNA-Seq analysis of potential lncRNAs and genes for the anti-renal fibrotic effect of norcantharidin. Journal of cellular biochemistry.

[17] Huang T, Wang G, Yang L (2017). Transcription Factor YY1 Modulates Lung Cancer Progression by Activating lncRNA-PVT1. DNA and cell biology.

[18] Chae YM, Park KK, Lee IK (2006). Ring-Sp1 decoy oligonucleotide effectively suppresses extracellular matrix gene expression and fibrosis of rat kidney induced by unilateral ureteral obstruction. Gene therapy.

[19] Mehta N, Zhang D, Li R (2019). Caveolin-1 regulation of Sp1 controls production of the antifibrotic protein follistatin in kidney mesangial cells. Cell communication and signaling: CCS.

[20] Tang H, Fan D, Lei CT (2016). MAD2B promotes tubular epithelial-to-mesenchymal transition and renal tubulointerstitial fibrosis via Skp2. Journal of molecular medicine (Berlin, Germany).

[21] Cai J, Liu Z, Huang X (2020). The deacetylase sirtuin 6 protects against kidney fibrosis by epigenetically blocking β-catenin target gene expression. Kidney international.

[22] Li X, Pan J, Li H (2020). DsbA-L mediated renal tubulointerstitial fibrosis in UUO mice. Nature communications.

[23] Zeng D, Xiao Z, Xu Q (2020). Norcantharidin protects against renal interstitial fibrosis by suppressing TWEAK-mediated Smad3 phosphorylation. Life sciences.

[24] Ruiz-Ortega M, Rayego-Mateos S, Lamas S (2020). Targeting the progression of chronic kidney disease. Nature reviews Nephrology.

[25] Edeling M, Ragi G, Huang S (2016). Developmental signalling pathways in renal fibrosis: the roles of Notch, Wnt and Hedgehog. Nature reviews Nephrology.

[26] Djudjaj S, Boor P (2019). Cellular and molecular mechanisms of kidney fibrosis. Molecular aspects of medicine.

[27] Yang Z, Jiang S, Shang J (2019). LncRNA: Shedding light on mechanisms and opportunities in fibrosis and aging. Ageing research reviews.

[28] Vellingiri B, Iyer M, Devi Subramaniam M (2020). Understanding the Role of the Transcription Factor Sp1 in Ovarian Cancer: from Theory to Practice. International journal of molecular sciences.

[29] Ito T, Azumano M, Uwatoko C (2009). Role of zinc finger structure in nuclear localization of transcription factor Sp1. Biochemical and biophysical research communications.

[30] Ito T, Kitamura H, Uwatoko C (2010). Interaction of Sp1 zinc finger with transport factor in the nuclear localization of transcription factor Sp1. Biochemical and biophysical research communications.

[31] Radhakrishnan R, Kowluru RA (2021). Long Noncoding RNA MALAT1 and Regulation of the Antioxidant Defense System in Diabetic Retinopathy. Diabetes.

[32] Geng W, Zhou G, Zhao B (2020). Liquiritigenin suppresses the activation of hepatic stellate cells via targeting miR-181b/PTEN axis. Phytomedicine.

[33] Kato M, Wang M, Chen Z (2016). An endoplasmic reticulum stress-regulated lncRNA hosting a microRNA megacluster induces early features of diabetic nephropathy. Nature communications.

[34] Van der Hauwaert C, Glowacki F, Pottier N (2019). Non-Coding RNAs as New Therapeutic Targets in the Context of Renal Fibrosis. International journal of molecular sciences.

[35] Vizcaíno C, Mansilla S, Portugal J (2015). Sp1 transcription factor: A long-standing target in cancer chemotherapy. Pharmacology & therapeutics.

[36] Safe S, Abbruzzese J, Abdelrahim M (2018). Specificity Protein Transcription Factors and Cancer: Opportunities for Drug Development. Cancer prevention research (Philadelphia, Pa).

