# Supplementary material for: NCTD Prevents Renal Interstitial Fibrosis via Targeting Sp1/lncRNA Gm26669 Axis
Source: Int J Biol Sci. 2021 Jul 25;17(12):3118–32. doi: 10.7150/ijbs.59195 (PMC8375230; doi:10.7150/ijbs.59195)
Supplement: Supplementary file 1 — Supplementary figures. [file ijbsv17p3118s1.pdf]

Figure legends

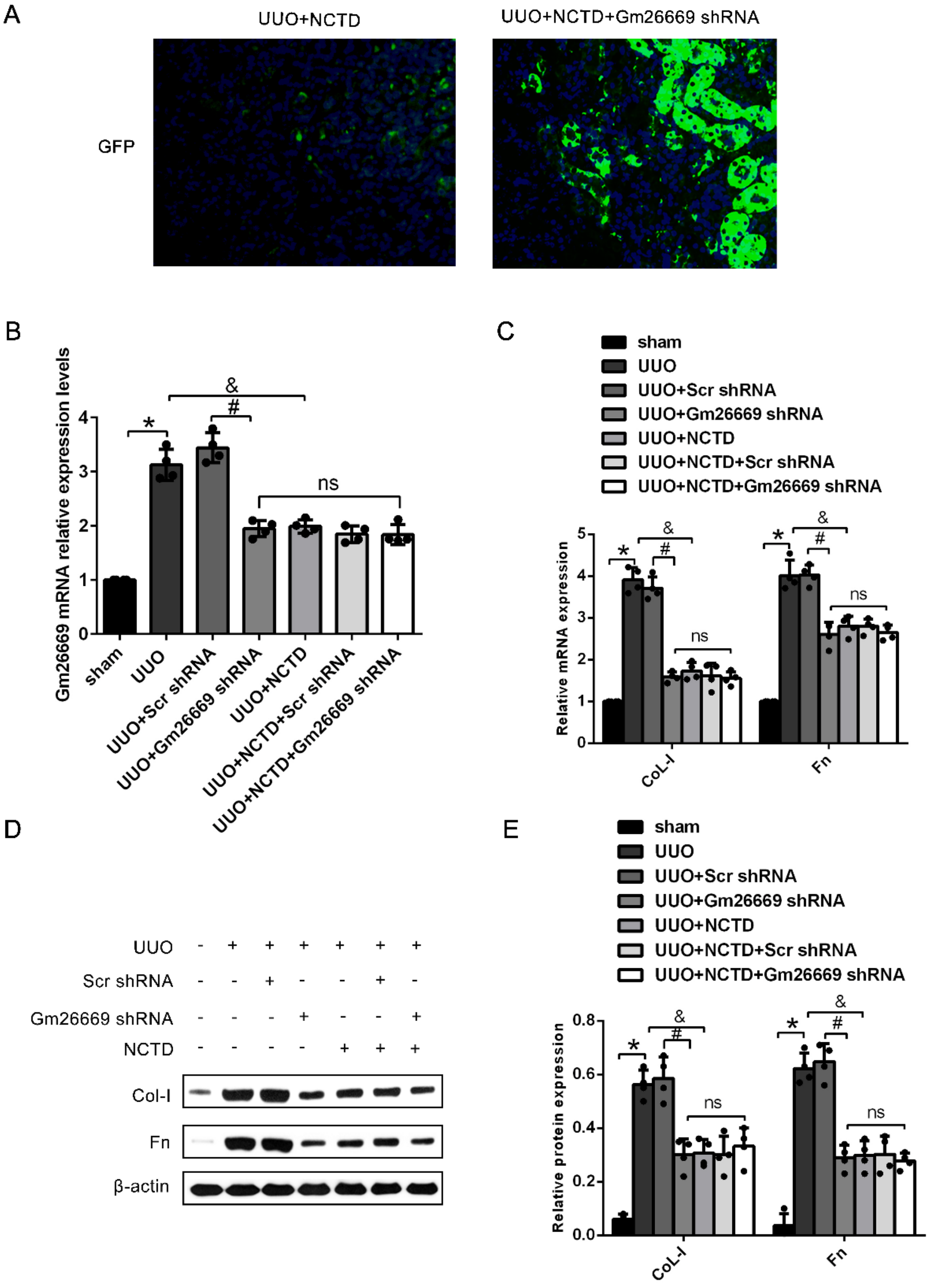

Supplementary Figure 1. The effects of NCTD on the deposition of ECM in the kidneys of UUO mice injected with Gm26669 shRNA.

(A) The IF staining of GFP in the kidneys of UUO mice injected with Gm26669 shRNA

and treated with NCTD (400×). (B) qRT-PCR analyses of Gm26669 expression in the kidneys of UUO mice injected with Gm26669 shRNA and treated with NCTD (C-E) qRTPCR, Western blotting, and densitometric analyses of CoL-I and Fn expression levels in the kidneys of UUO mice injected with Gm26669 shRNA and with NCTD treatment.. *P<0.05, #P<0.05, &P<0.05, ^P<0.05; data are presented as the mean ± SEM (n=4).

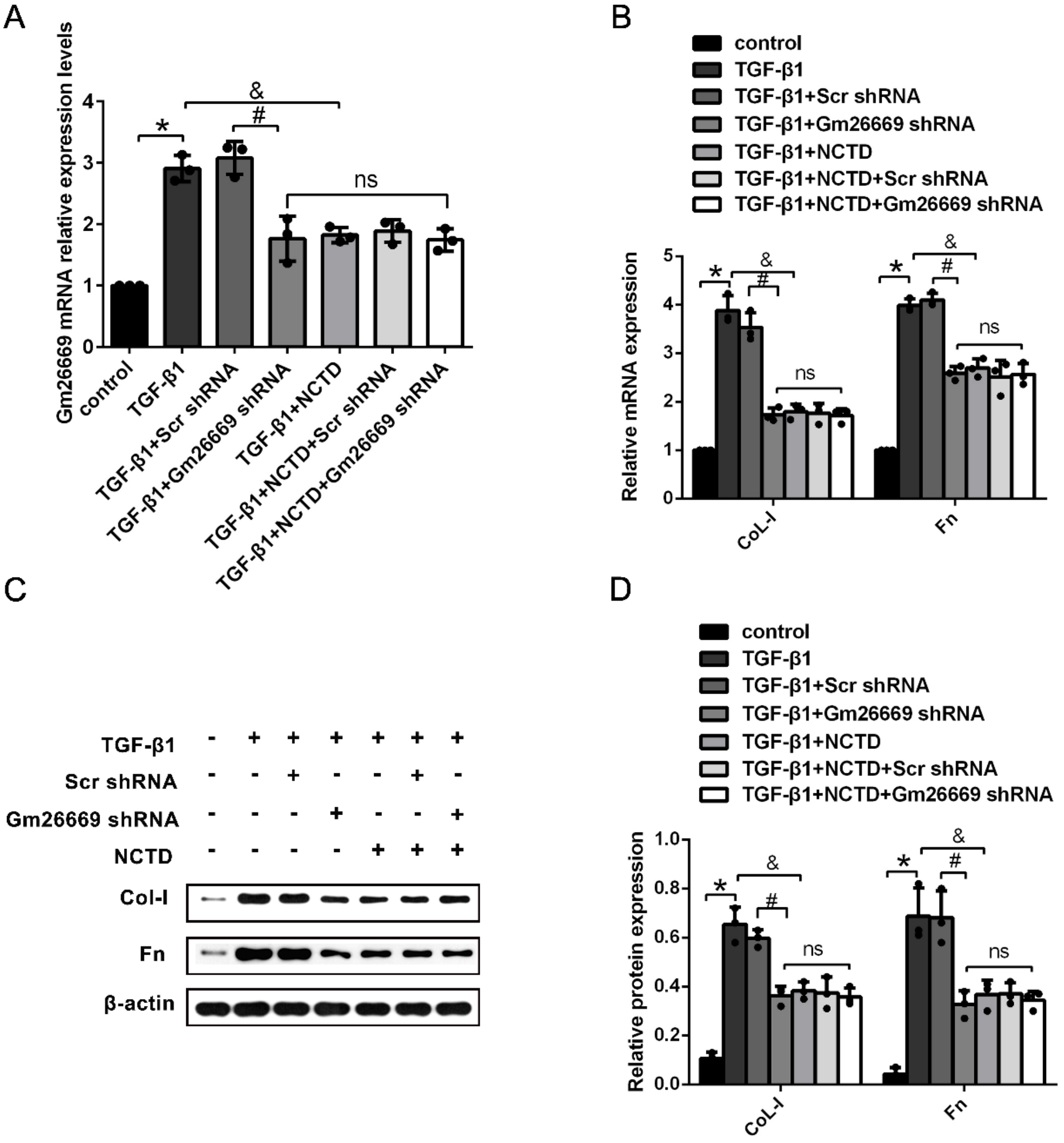

Supplementary Figure 2. The effects of NCTD on ECM deposition in BUMPT cells with Gm26669 shRNA transfection.

(A) qRT-PCR analyses of Gm26669 expression in TGF-β1-stimulated BUMPT cells following the transfection of Gm26669 and NCTD treatment. (B-D) Western blotting and densitometric analyses of CoL-I and Fn expression levels in TGF-β1-stimulated BUMPT cells following transfection with Gm26669 shRNA and NCTD treatment. *P<0.05, #P<0.05, &P<0.05, ^P<0.05; data are presented as the mean ±SEM (n=4).
